# Unsupervised meta-analysis on chemical elements and atomic energy prediction: A case study on the periodic table

**DOI:** 10.1016/j.heliyon.2024.e37240

**Published:** 2024-08-30

**Authors:** Brahim Belahcene

**Affiliations:** Abou Bekr Belkaid University, Tlemcen, Algeria

**Keywords:** Artificial classification, Chemical elements, Periodic table, Atomic energy, DFT, LDA, Data mining, PCA

## Abstract

This paper presents an artificial classification and atomic energy correlation analysis of the chemical components. The choice of data mining is due to its robustness, which can explore intrinsic or hidden relationships between chemical components and their properties. The Mendeleev table is conceivably the earliest example of the data analysis technique in materials science. However, the classical periodic table represents the arrangement of chemical elements based on particular periodicities, which has the issue of property progression for a few chemical components. In this investigation, we utilized one of the unsupervised data mining methods (principal component analysis) to explore knowledge from the chemical components database based on all the prepared properties. The main objective is to make an artificial classification of chemical components depending on their accessible physical and energetic properties. The results revealed the effectiveness of the data mining method in appreciating the relationships between the variables and properties that offer a new approach to seeing a Mendeleev table. The final step of this work highlights the significance of predictive polynomials that permit the scientific community to make atomic total energy predictions for each chemical component, from helium to lawrencium.

## Nomenclatures

V1: Atomic number (Z)
V2: Atomic weight (A)
V3: The states of oxidation.
V4: Electronegativity (χ) (Allred and Rochow)
V5: First ionization energy (I.E) (eV)
V6: The lattice parameter (a) (pm)
V7: The number of atoms per cell (N)
V8: The molar volume (Vm) (cm3/mol)
V9: Young's modulus (E) (GPa)
V10: Elastic compliances (S11) in the normal plane 11 (1/TPa)
V11: Elastic compliances (S44) in the normal plane 44 (1/TPa)
V12: Elastic compliances ((S12) in the normal plane 12 (1/TPa)
V13: stiffness in the normal plane (C11) in the normal plane11 (GPa)
V14: stiffness in the shear plane (C44) in the normal plane 44 (GPa)
V15: Thermal conductivity (*k*) (W/(m K))
V16: Molar heat capacity (Cp) (J/(mol K)) At 298 K.
V17: Standard entropy (S°) (J/(mol K)) At 298 K and 100 kPa.
V18: The Enthalpy difference H298 − H0 (ΔH°) (kJ/mol)
V19: The melting temperature (Tm) (K)
V20: The change in enthalpy at fusion (ΔH_f) (J/(mol K))
V21: The boiling temperature (T_b) (K)
V22: Change in enthalpy ΔHb (kJ/mol)
Etotal Total energy (Hartree)
Ec Kinetic energy (Hartree)
E e−nucl, Een Energy of Coulomb interaction (Hartree)
E ex-corre Exchange, and correlation energy (Hartree)
PC1 First principal axis (Eigenvalue) (λ) of news components.
PC2 Second principal axis (Eigenvector) (v) of news components.
[PC1PC2] Cartesian plane (vector cross product of PC1 and PC2)

## 1 Introduction

The disclosure of chemical components and the reasoning behind their classification started decades prior. The systematic exploration of fundamental particles and atomic physics has evolved over time as detailed in Appendices A and B (the organized literature network and bibliographic information map).

The early stage developed based on the scientific work results known since 1800. At that point, in 1743–1794, Lavoisier portrayed thirty-three substances and their chemical composition [1]. Along these lines, in 1807–1808, Davy separated sodium, potassium, barium, strontium, and calcium through electrolysis [2]. Davy and Döbereiner characterized the set of three laws to take note of the relationship between bromine, iodine, chlorine, sulfur, tellurium, and selenium [3]. At the same period, Dalton described chemical components by their nuclear weight [4].

Subsequently, the notions introduction of physicochemical properties and the differentiation of molecules or atoms was done by Gay-Lussac in 1809 [5] and Avogadro in 1811 [6]. Dalton did not adopt the Latin names of substances as standard symbols for chemical elements, unlike the system proposed by Berzelius in 1814 [7]. The idea of an atomic mass system and the molecule was announced in 1860 at the primary worldwide chemistry congress in Karlsruhe [8]. In 1867, Dmitry Mendeleev was granted as a teacher of mineral chemistry at the College of St. Petersburg after accomplishing his work on the density of gasses and the spectroscope of Gustav Kirchhoff [[8], [9], [10], [11]], where he classified vertically the chemical components as of now decided in his time according to atomic number and mass. Within the same period, Mendeleev anticipated the properties of a few lost chemical components from their surroundings utilizing the triad law. A review of form illustrations of the periodic elements over 100 long times was detailed by Foster [12] in 1985; the graphic illustration as a spiral and blocks of chemical elements was described by Imyanitov [13] in 2016; and the philosophy and the presentation of quantum mechanics and the essential concepts of chemical components were detailed by Scerri [14] in 2020; Pyykkö [16] in 2019; Imyanitov [13] in 2016; van Spronse [15] in 1971).

The identification of the charges of the atomic nucleus, as well as the highlighting of missing chemical components, was carried out by Henry Moseley utilizing the frequencies of X-rays [17]. The description of chemical bonding utilizing electronegativity as a property can be based on the thermochemical scale first proposed by Pauling [18]. Tantardini and Oganov proposed two approaches for determining bond dipole moment. First, they suggested predicting it directly from the thermodynamic electronegativity scale [19]. Second, they considered it a spectroscopic parameter, building on Mulliken's initial concept [20] and Rahm and Hoffman later improved upon this [21]. The periodic table organizes chemical elements based on recurring patterns. There are currently 118 known elements and can be expanded with new discoveries without violating atomic physics laws that maintain nuclear stability. The classical arrangement of chemical elements defines that the chemical components have the same valence number band of electrons grouped within the same columns.

Additionally, horizontal categorization is based on expanding numbers of electrons from top to bottom, then from left to right. The Mendeleev table is arguably the first example of data analysis in materials science. This research employs unsupervised classification, specifically principal component analysis (PCA), for statistical analysis of chemical elements.

### 1.1 Philosophical questions about chemical elements

The questions that ought to be inquired before plunging into the heart of the matter will open a philosophical and logical intellect almost the beginning, investigation, and inquiry concerning thechemical components in our universe.

➢ What is the distribution form of chemical elements, based on many properties?
➢ Do the chemical elements follow a logical law based on a few properties?
➢ Can we improve the properties of materials or create new materials or genomic materials based on hidden, strong relationships between elements?
➢ What is the distribution of chemical elements based on atomic energies?
➢ What is the impact of the energy of exchange and correlation on the trend of the distribution of chemical elements?

It is critical to note that the exchange-correlation term comes when considering the DFT strategy created by Kohn & Pretense [41]. This term remains applicable to post-Hartree-Fock methods such as CCSD(T), which excel at describing isolated atoms.

## 2 Methods

### 2.1 Unsupervised meta-analysis and nonlinear technical regression description

The arrangement is a crucial step in data analysis; it comprises object groups of a data set into homogeneous classes [22,23]. Within the present study, I utilized data mining methods to estimate the potential forecast of the chemical components. Data mining emerged in the 1990s to extract information from large databases [[24], [25], [26]]. PCA's core concept is to reduce the dimensionality of data matrices. This reduction is only possible if the initial variables are not independent and have non-zero correlation coefficients.

The initial variables will be new variables such as principal components, estimated by linear combinations. Principal component analysis (PCA) determines the orthogonal eigenvectors and their corresponding eigenvalues of the scatter matrix of the original variables; the orthogonal eigenvectors are used to construct the principal components, and the eigenvalues are the variances of the principal component analysis [[24], [25], [26], [27], [28], [29], [30], [31]].

In this work, we used PCA to display hidden relationships between elements and their properties. The polynomial regression used in the present research predicts the relationship between dependent and independent variables [32]. Able to assess the nonlinear relationship of factors by expanding the range of polynomial terms [33]. Some researchers have applied extensive polynomial and trigonometric regression to characterize curvilinear relationships [34]. Predictive modeling employs sophisticated research methodologies, including artificial neural networks. ANN performance can be assessed using the coefficient of determination (R^2^), mean squared error (MSE), and mean absolute error (MAE), as well as Jacobian calculations (Levenberg-Marquardt) [42]. Consequently, nonlinear regression was used in the present first stage of research to predict the relationship between electronic properties, total energies, and atomic number from helium to lawrencium.

## 3 Results and physical interpretation

**Fig. 1:**
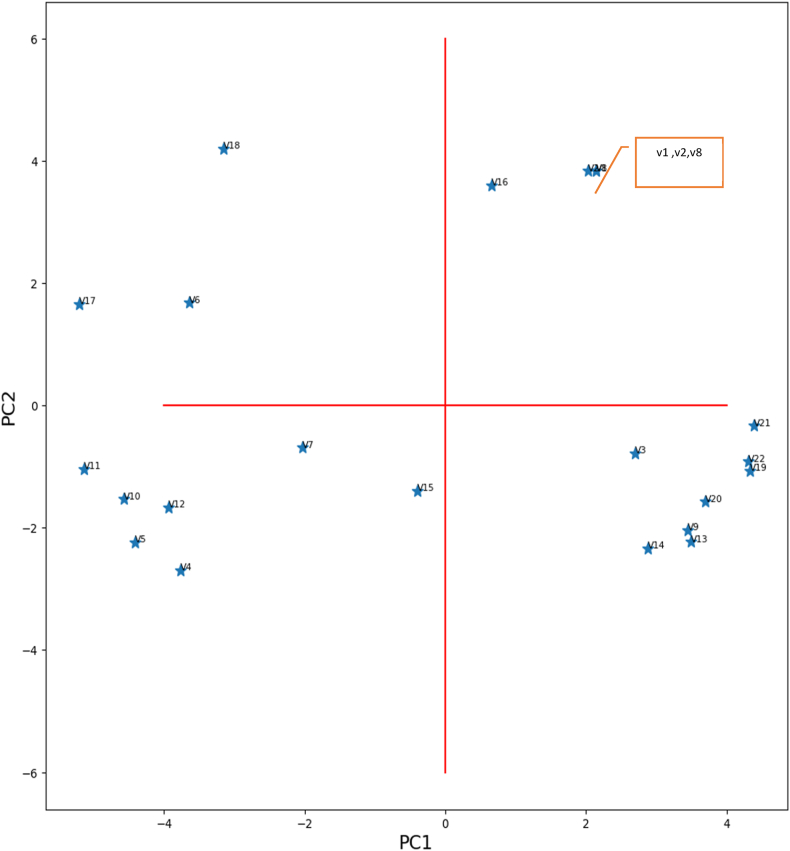
Scores plot -The correlation between physical properties.

**Fig. 2:**
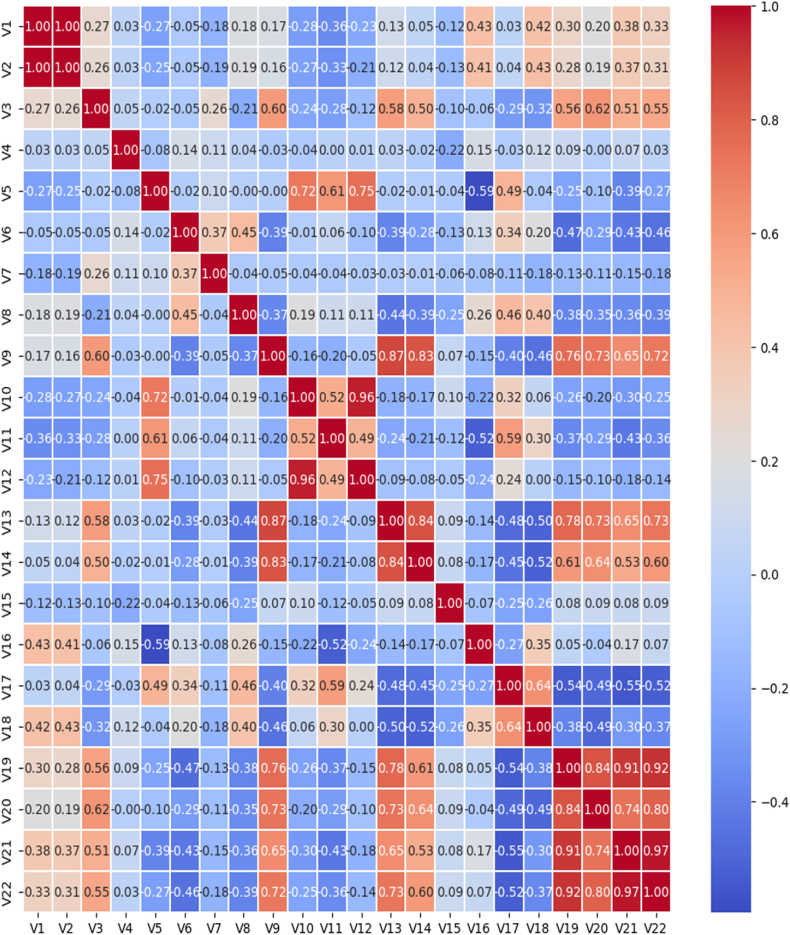
Heat map correlation matrix.

**Fig. 3:**
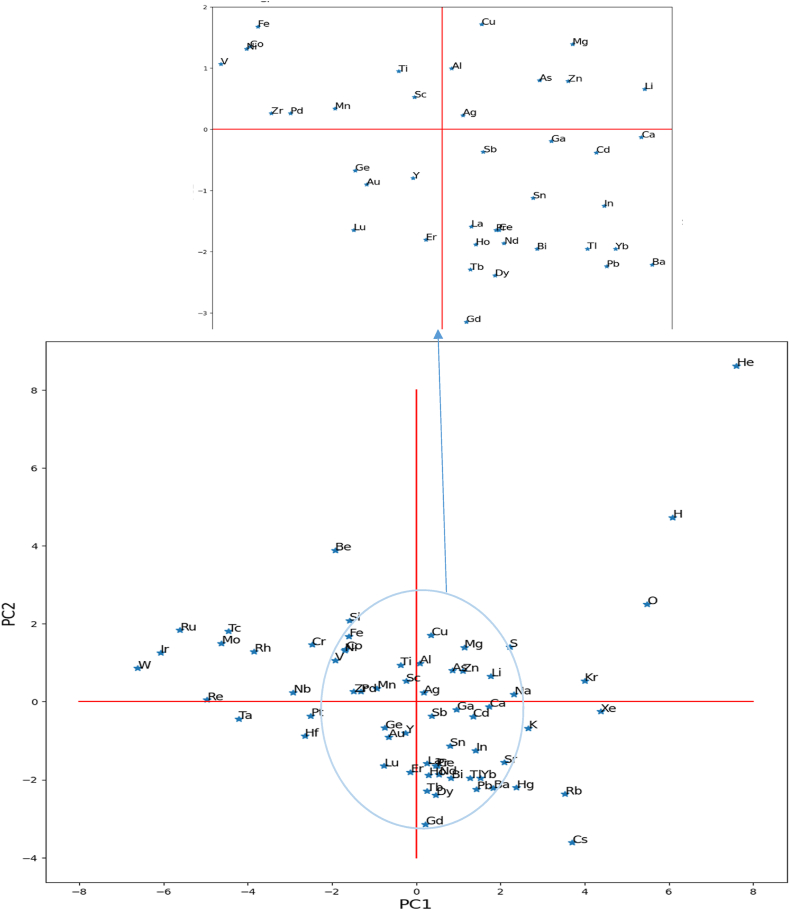
Two-dimensional variable (loading plot). Classification of the elements of the periodic table according to physical properties.

Fig. 1 presents the diagram of properties (scores plot), then Fig. 3 shows the relationships of variables (loading plot) based on experimental data [35]. These results allow us to detect the correlations between the twenty-two physical properties of the elements, renamed by [V1, V2 … V22] to get better visibility of the diagram (see Fig. 2).

V1 correlated to V2: Atomic number and mass are directly related when an atom's mass equals the combined mass of its neutrons and protons.

V2 correlated to V8: The atomic weight and the molar volume are correlated, which is logical since the molar volume is a function of density and weight.

V1, V2, and V8 correlated to V16: Atomic number, atomic weight, and molar volume influence molar heat capacity. This relationship can be explained as follows: Heat capacity measures a material's ability to absorb heat. The phonons are the primary mechanism through which this heat energy is absorbed.

V19 and V20 correlated to V21 and V22: The melting and boiling temperatures correlate to the change in enthalpy at fusion and boiling state. This last quantity expresses the energy supplied so that the material passes from the solid to the liquid state (phases behaviors).

V13 and V14 correlated to Var9: The elastic constants C11 and C12 and the young modulus have relationships. The two constants define the material response to deformation on the atomic scale.

The Young modulus is the macroscopic response of the material to tensile deformation; certainly, macroscopic mechanical behavior properties are expressions of the displacement of atoms on a smaller scale upon applying external stress.

V4 correlated to V5: Electronegativity and the energy of the first ionization are correlated; an electronegative material tends to attract electrons from other atoms in a bond. For stronger atomic binding, the electrons of the last orbit are strongly linked and are very difficult to exit, expressed by high ionization energy.

V17, V18, and V6 are correlated: The entropy has a relationship with the lattice parameter and the molar volume; it implies that the increase in molar volume increases the disorder in the system.

V10 correlated to V11 and V12: The elastic compliances S11, S12, and S44 have a relationship; this is consistent as long as all these quantities are related to how the structure varies when the external stress is applied. In other words, they all arise from the interatomic bond. On the other hand, the anti-correlations between some variables are the following:

V9 has an inverse correlation to V6 and V8: Young's modulus has an inverse correlation to the lattice parameter and the molar volume; it is known from experience that Young's modulus is proportional to the compression module that measures the lattice compressibility. It is difficult to compress the atomic volume when the lattice parameter and the molar volume decrease, and the volume per atom, also decreases; consequently, the atom's possibility of bringing closer together decreases. It explains why the network parameter decreases the compression module as much and why Young's modulus increases.

Consequently, the decrease of electrons implied less energy required to tear them away (first ionization energy) decreases. Also, when the number of electrons increases, the electronegativity decreases, and its' nucleus doesn't attract electrons from adjacent atoms more strongly [39,40].

### 3.1 Dimensional representation of the variable diagram

Fig. 3 reveals the spatial distribution of elements in the cartesian plane [PC1PC2]. So it is seen that helium, hydrogen, oxygen, and some rare gases (Xe, Kr …) are visible on the side of the diagram. Then, tungsten occupies a particular position distinguished by some metallic elements (Ir, Ru …) [39,40]. However, the other components have a strong correlation, with the significant part concentrated in the center of the curve, which required us to zoom certain clouds for better readability.

As seen in this form, some chemical elements condense at the same center of gravity. Indeed, as much as the property graph reflects physical correlations, the more the illustration of the variables is condensed.

The perpendicular direction allows the visualization of the chemical components. Therefore, it remains to note that some distribution of elements in the graph shows a grouping according to the closest and similar properties; the large number of properties used in this analysis is likely responsible for the variance in the results and the distribution of the chemical elements [39,40].

### 3.2 Atomic energy and electronic system correlations analysis

The elements and their properties refer to the database based on LDA calculations [36] as the total energy, coulombian, kinetic, exchange, and correlation energy arranging from Hydrogen to Uranium.

**Fig. 4:**
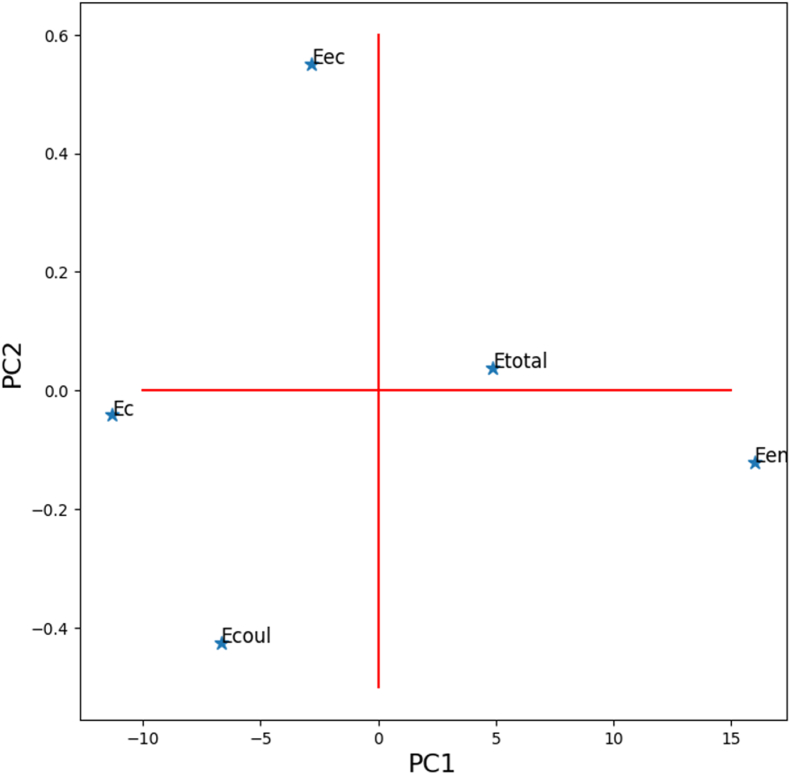
The correlation between electronic properties - Scores plot.

**Fig. 5:**
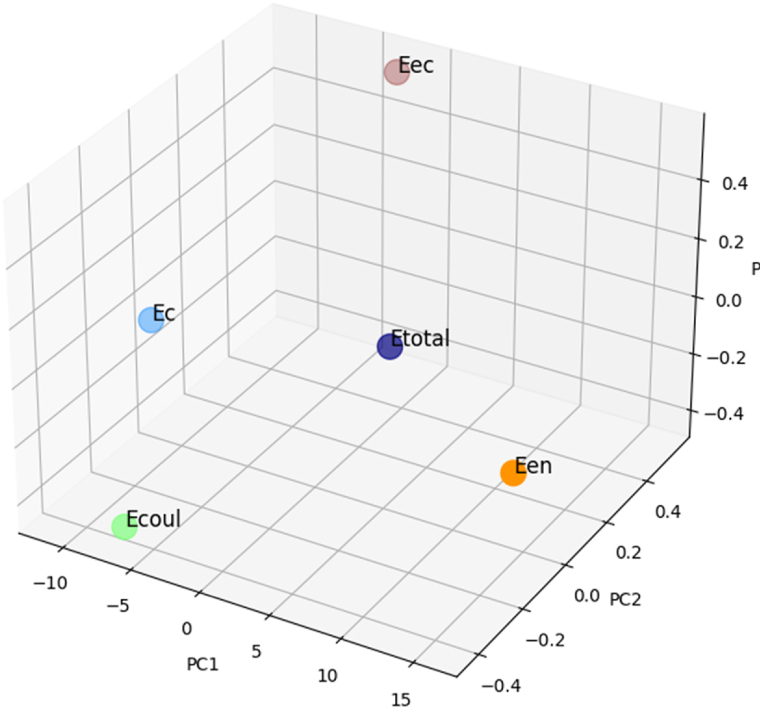
3D visualization of electronic properties correlation - Scores plot.

Fig. 4, Fig. 5 represent the properties plot (score plot); it is noticed that the atomics energies are distributed practically only along the axis PC1.

It is observed that the energy distribution is predominantly aligned with the PC1 axis. The electron nucleus electrostatic interaction energy and the total energy are on the positive side of PC1, while the kinetic energy and the electron electrostatic interaction energy are on the negative side of PC1 [39,40].

**Fig. 6:**
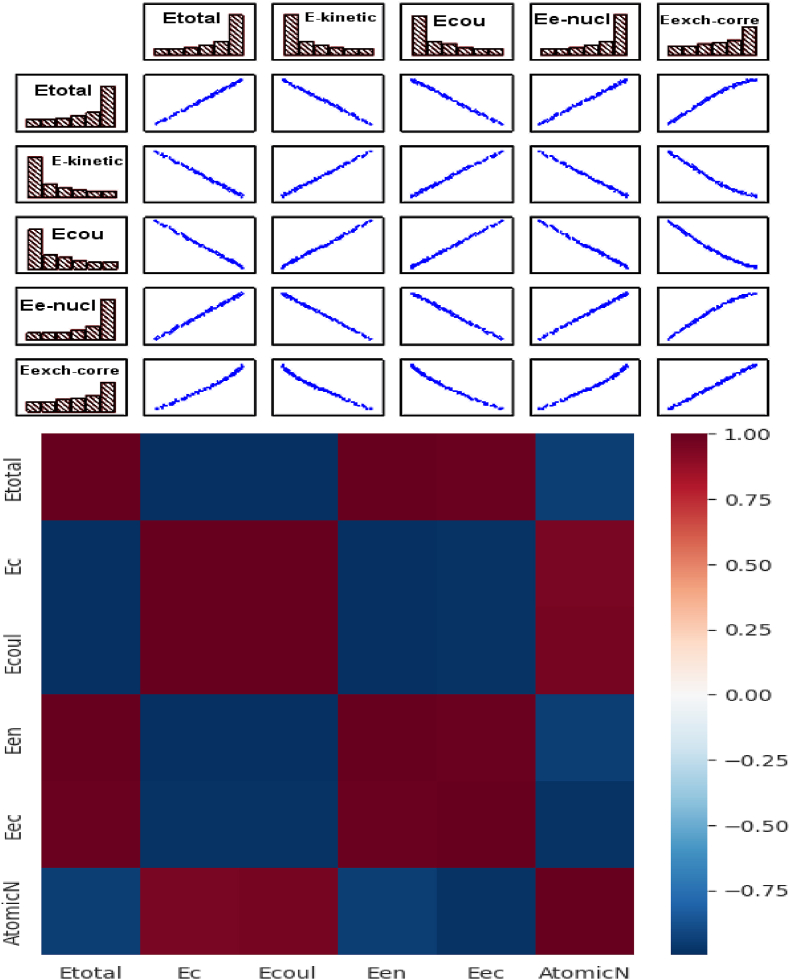
Heat map correlation matrix of energies.

**Fig. 7:**
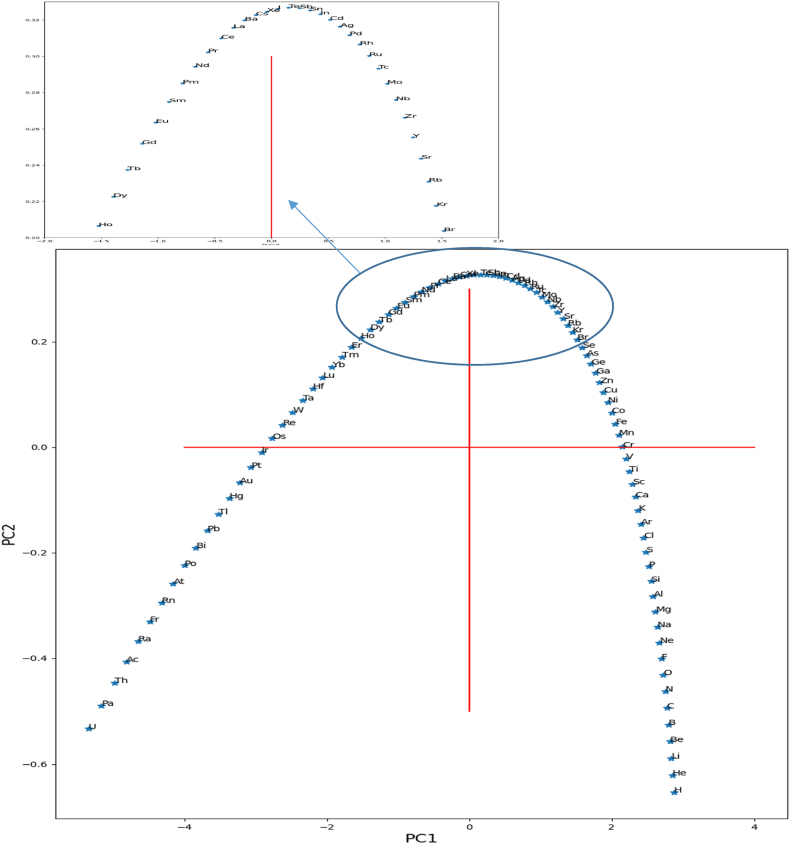
The correlation between elements - Loading plot, distribution of the elements according to atomic energies "LDA".

The energy of exchange and correlation is also on the right side, but with a few components according to PC2. The matrix shown in Fig. 6 illustrates correlation types between energies (see Table 1). Fig. 7 represents the diagram of the variables (loadings plot); a few areas have zoomed in to show the element layout scatter plot. As shown in Fig. 7, the periodic table is aligned according to its atomic number, starting from hydrogen to uranium in the opposite direction of the PC1 axis. Also, according to PC2, they form a kind of Gauss curve in the positive direction of the PC2 axis. It can be explained as follows: The negative direction of the PC1 axis is correlated with the electron-electron interaction energy and kinetic energy of electrons, then the elements with more electrons move in this direction [39,40].

**Table 1:** The correlation matrix of energies (Etotal, Ec, E e−nucl, E ex-corre).

	Etotal	Ec	Ecoul	Ee-nucl	E exch.corre
Etotal	1.00000	−1.00000	−0.999847	0.999997	0.989770
Ec	−1.00000	1.00000	0.999847	−0.999997	−0.989760
Ecoul	−0.99985	0.99985	1.000000	−0.999885	−0.991017
Ee-nucl	1.00000	−1.00000	−0.999885	1.000000	0.989850
E exch.corre	0.98977	−0.98976	−0.991017	0.989850	1.000000

**Fig. 8:**
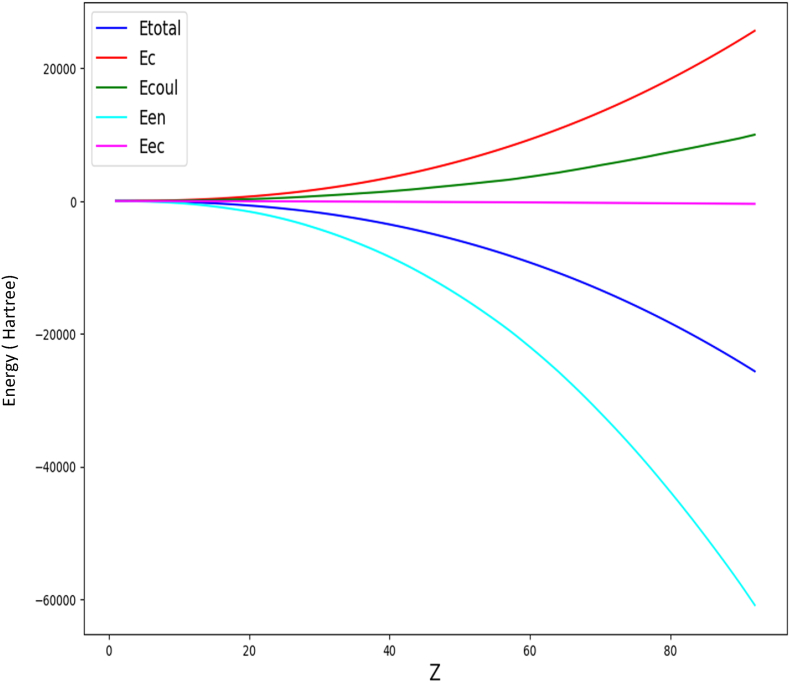
Atomic energy distributions (Hartree).

The distribution curve of the elements also reveals trends for the heaviest and lightest elements. For heavy elements, Coulomb interaction energy dominates over exchange and correlation energy due to the large number of electrons, as depicted in Fig. 8. Conversely, for light elements, there are insufficient electrons for significant exchange and correlation energy. Consequently, the elements in the middle of the distribution are more influenced by exchange and correlation energy, causing an upward shift in their positions.

(1) E Total Predicted = (p1*z^10^ +p2*z^9^ +p3*z^8^ + p4*z^7^ +p5*z^6^ + p6*z^5^ +p7*z^4^ + p8*z^3^ + p9*z^2^ +p10*z + p11) *10^^5^

P1 = 3.67E-20; P2 = −2.00E-17; P3 = 4.51E-15; P4 = −5.51E-13; P5 = 3.99E-11; P6 = −1.76E-09; P7 = 4.91E-08; P8 = −1.07E-06; P9 = −6.11E-06; P10 = 5.46E-06; P11 = −6.44E-06.

**Fig. 9:**
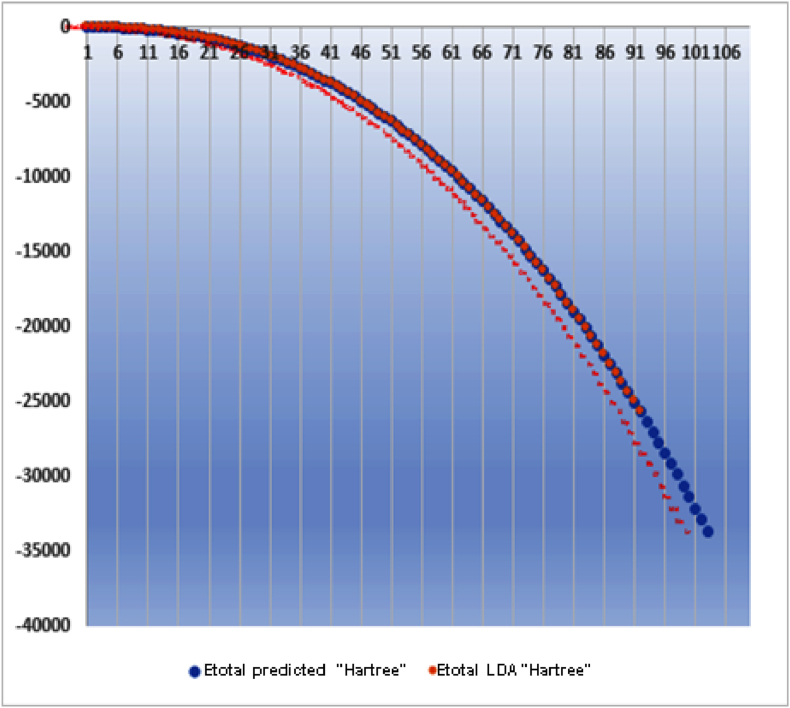
The polynomial of total atomic energy.

Table 2 presents the total energy calculated using polynomial equation (1) depicted in Fig. 9. Within the error margin, these results align closely with the NIST Standard Reference Database [35,36] for elements ranging from helium to lawrencium [40].

**Table 2:** 

z	Etotal predicted Hartree	Etotal LDA^31-32^ Hartree	SD
93	−26374.2130385254	−26325.4386967855	0.18 %
94	−27054.7117761243	−27002.6683849033	0.19 %
95	−27745.9078872578	−27689.6834855652	0.20 %
96	−28448.1151881729	−28386.4092359141	0.22 %
97	−29161.6974796868	−29093.9325964725	0.23 %
98	−29887.0721625673	−29811.2722013778	0.25 %
99	−30624.7135474639	−30538.8250815148	0.28 %
100	−31375.1557599997	−31276.6433895199	0.31 %
101	−32138.9951310089	−32024.7791035710	0.36 %
102	−32916.8919507090	−32783.2840569287	0.41 %
103	−33709.5714538646	−33551.5868267897	0.47 %

## 4 Conclusions

This study constructs a database based on the physical properties of chemical elements. Principal Component Analysis (PCA) is employed to analyze this database and visualize the results using score and loading plots to identify relationships among the elements' properties. Therefore, principal component analysis (PCA) is one of the unsupervised techniques used for analyzing a database of chemical elements' properties, such as well as physicochemical, mechanical, thermal, and electronic properties, such as kinetic, electrostatic, total, and exchange energy. This approach carries out correlation energy from hydrogen until atomic number ninety-two, which makes a new way of viewing the table of Mendeleev, and atomic total energy predictions. Therefore, it revealed that kinetic and electron-electron interaction energy characterize heavy elements. On the other hand, electron-nucleus interaction energy and the total energy by the light components. Moreover, the study found that exchange and correlation energy significantly influence the element distribution in the loading plot, requiring further physical interpretation.

## Data availability statement

The data used in this research are available upon reasonable request from qualified researchers interested in this field. Please contact us [belahcene.brahim@gmail.com] for details and access procedures.

## CRediT authorship contribution statement

**Brahim Belahcene:** Writing – review & editing, Writing – original draft, Visualization, Validation, Supervision, Software, Resources, Project administration, Methodology, Investigation, Funding acquisition, Formal analysis, Data curation, Conceptualization.

## Declaration of competing interest

The authors declare that they have no known competing financial interests or personal relationships that could have appeared to influence the work reported in this paper.

## Figures and Tables

**Fig. A:**
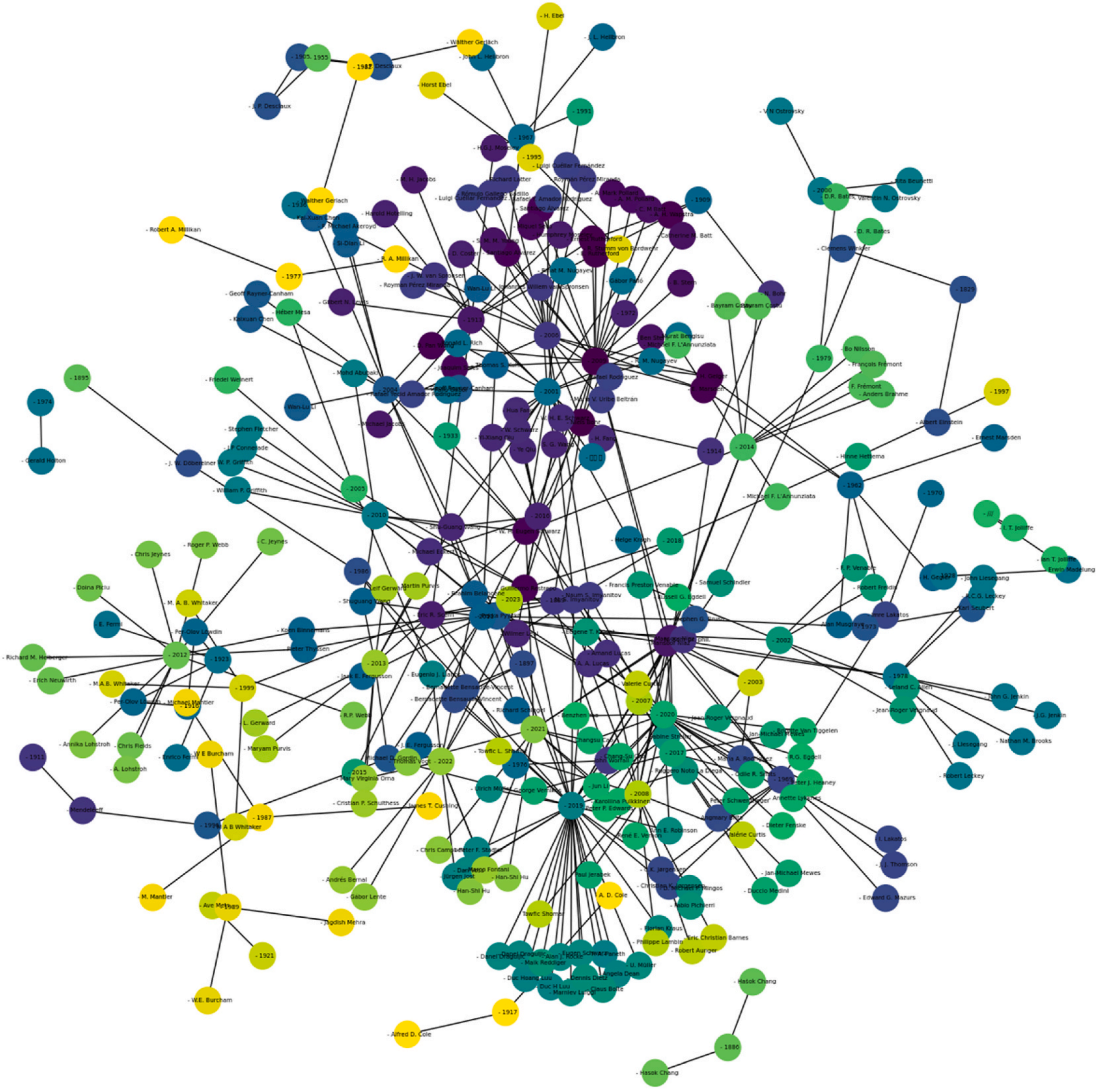
Network literature connectivity

**Fig. B:**
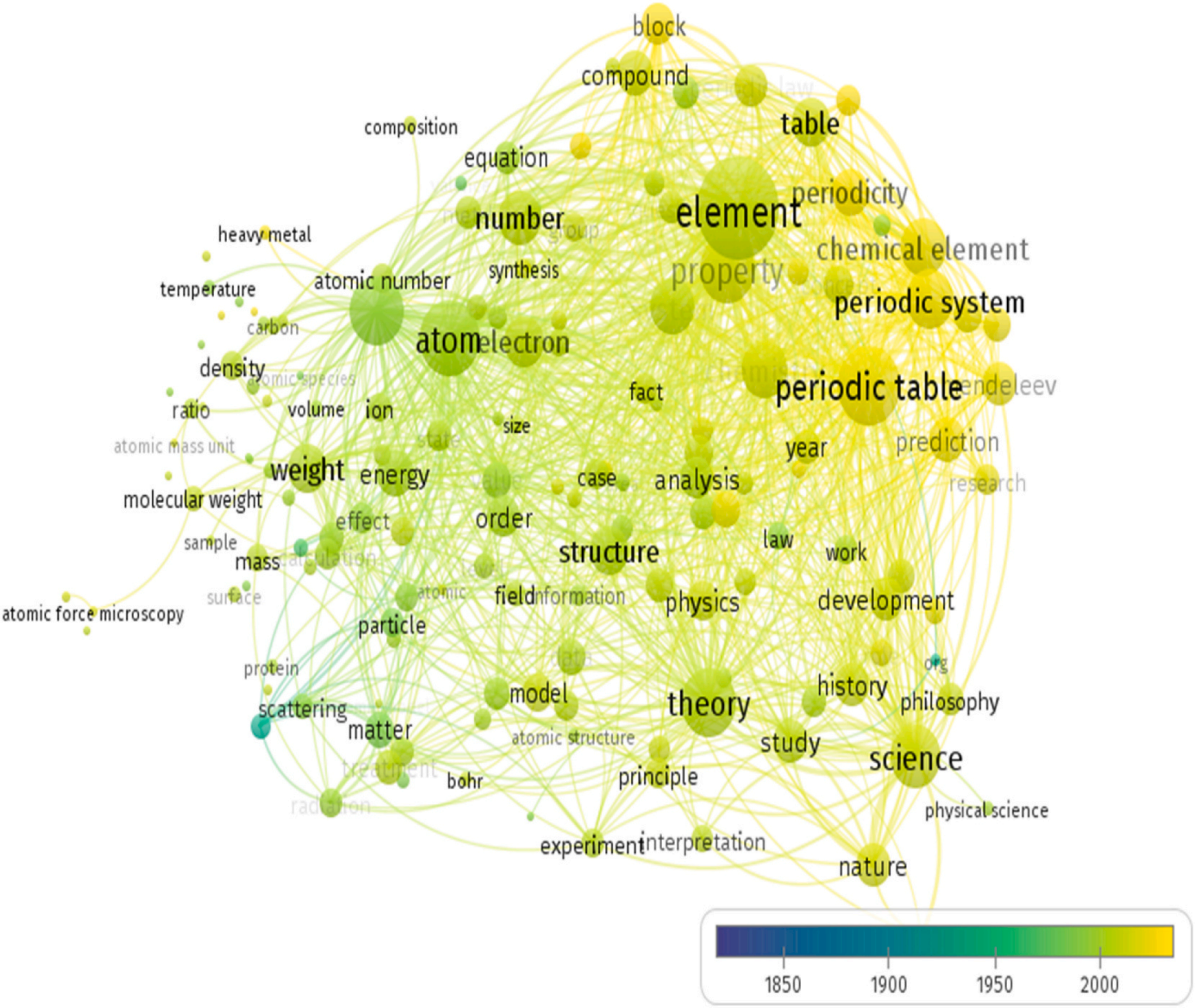
Bibliographic data map
